# Dramatic expansion of the black widow toxin arsenal uncovered by multi-tissue transcriptomics and venom proteomics

**DOI:** 10.1186/1471-2164-15-366

**Published:** 2014-06-11

**Authors:** Robert A Haney, Nadia A Ayoub, Thomas H Clarke, Cheryl Y Hayashi, Jessica E Garb

**Affiliations:** Department of Biological Sciences, University of Massachusetts, Lowell, MA 01854 USA; Department of Biology, Washington and Lee University, Lexington, VA 24450 USA; Department of Biology, University of California, Riverside, CA 92521 USA

**Keywords:** RNA-Seq, Latrotoxins, Venom, Mass spectrometry, Transcriptomics, Spider

## Abstract

**Background:**

Animal venoms attract enormous interest given their potential for pharmacological discovery and understanding the evolution of natural chemistries. Next-generation transcriptomics and proteomics provide unparalleled, but underexploited, capabilities for venom characterization. We combined multi-tissue RNA-Seq with mass spectrometry and bioinformatic analyses to determine venom gland specific transcripts and venom proteins from the Western black widow spider (*Latrodectus hesperus)* and investigated their evolution.

**Results:**

We estimated expression of 97,217 *L. hesperus* transcripts in venom glands relative to silk and cephalothorax tissues. We identified 695 venom gland specific transcripts (VSTs), many of which BLAST and GO term analyses indicate may function as toxins or their delivery agents. ~38% of VSTs had BLAST hits, including latrotoxins, inhibitor cystine knot toxins, CRISPs, hyaluronidases, chitinase, and proteases, and 59% of VSTs had predicted protein domains. Latrotoxins are venom toxins that cause massive neurotransmitter release from vertebrate or invertebrate neurons. We discovered ≥ 20 divergent latrotoxin paralogs expressed in *L. hesperus* venom glands, significantly increasing this biomedically important family. Mass spectrometry of *L. hesperus* venom identified 49 proteins from VSTs, 24 of which BLAST to toxins. Phylogenetic analyses showed venom gland specific gene family expansions and shifts in tissue expression.

**Conclusions:**

Quantitative expression analyses comparing multiple tissues are necessary to identify venom gland specific transcripts. We present a black widow venom specific exome that uncovers a trove of diverse toxins and associated proteins, suggesting a dynamic evolutionary history. This justifies a reevaluation of the functional activities of black widow venom in light of its emerging complexity.

**Electronic supplementary material:**

The online version of this article (doi:10.1186/1471-2164-15-366) contains supplementary material, which is available to authorized users.

## Background

Venomous taxa have evolved many times within the metazoa [1], and occur in both vertebrates and invertebrates. The venoms these diverse taxa produce are chemically complex and play key roles in organismal ecology, functioning in both predation and defense. Molecules contributing to the toxicity of venom are the focus of sustained effort aimed at characterizing their physiological roles and biochemical action, given their potential in pharmacological and biomedical applications [2]. Venom toxins are often members of large gene families, and the study of their evolution can illuminate the roles of gene duplication, convergence and positive selection in generating the functional diversity of venoms [3]. Determining the molecular diversity of venoms is the necessary first step in this process, yet few studies have utilized large scale approaches for venom characterization.

Spiders (Order Araneae) are the most species-rich venomous clade, with >44,000 described species [4], the overwhelming majority of which are venomous. Estimates of the number of unique venom peptides and proteins produced by members of this clade range from 1.5 - 20 million [5–7], significantly more than are estimated from other major clades of venomous invertebrates such as scorpions and cone snails [8, 9]. The venoms of some spiders have been extensively studied, largely due to the potential for isolating novel insecticidal toxins [7], and reasons of direct medical concern [10–13]. However, past work has focused on a small fraction of total spider species, and much of the molecular diversity of spider venoms remains to be discovered.

Spider venom proteins characterized to date belong to several different broad classes: enzymes (such as proteases, phospholipases and hyaluronidases), small linear cytolytic peptides, and neurotoxins with differing functionality and size range [7]. The most commonly documented form of spider neurotoxin is a small (<15 kDa), disulfide-rich peptide. The disulfide bonds give rise to one of three typical structural motifs, the disulfide-directed β-hairpin, the Kunitz motif, or the inhibitor cystine knot (ICK), the last of which appears to be the most common amongst studied spider venoms [14]. The compact structure of ICK peptides renders them highly resistant to the actions of proteases in envenomated organisms, contributing to their efficacy [15]. Different ICK peptides specifically target different ion channels in the nervous system [11], and diverse sets of these peptides can occur within the venom of even a single species [14, 12], acting synergistically with one another and with small linear peptides [14, 16, 17] in a manner similar to the “toxin cabals” of cone snails [18].

The most prominent exception to this venom small-molecule (<15 kDa) dominance occurs in the black widow spiders (genus *Latrodectus,* family Theridiidae), which contain multiple large (>130 kDa) neurotoxic proteins known as latrotoxins, encoded by paralogous loci [19–26]. The best studied of the latrotoxins, α-latrotoxin, forms tetrameric complexes which bind to vertebrate presynaptic receptors and insert into neuronal membranes, forming calcium-permeable ion channels that stimulate massive neurotransmitter release [27]. α-Latrotoxin is also widely known as the causative agent of the extreme pain associated with black widow bites. Other functionally characterized latrotoxins differ in their phyletic specificity, affecting the nervous systems of only insects or crustaceans. Latrotoxin proteins are accompanied in the venom by low-molecular weight peptides called latrodectins (also known as α-latrotoxin associated LMWPs) that may enhance latrotoxin toxicity [20, 28], although they exhibit no toxicity themselves [29].

Given the large number of peptides and proteins remaining to be discovered in the venoms of spider species, next generation RNA sequencing (RNA-Seq) methods are particularly well suited for rapidly obtaining a comprehensive inventory of venom components, as well as an improved functional understanding of the venom gland. The high-throughput of next-generation sequencing allows for profiling of transcripts over a wide range of abundance [30], providing an accurate picture of differential expression across tissues within an organism. A multi-tissue approach allows for the identification of transcripts with highly biased expression in the venom gland, whose products are candidates for function in the venom as toxins, or in venom production. Venom gland specific sequences can then be subjected to bioinformatic and evolutionary analyses to discover novel toxins and to better understand their origins and the mechanisms generating their diversity. The insights provided by transcriptomic data can be greatly enhanced by proteomics approaches which permit a direct examination of the peptide and protein composition of venoms, typically with methods coupling liquid chromatography based separation to mass spectrometry [6]. These methods have begun to be applied to a range of species, leading to an expansion of the number of venom peptide and protein toxins known from arachnids [31, 32].

In this study we present an integrated set of multi-tissue transcriptomic and proteomic data from the Western black widow spider, *Latrodectus hesperus,* to investigate the composition and evolution of its venom. The venom of this species remains largely unexplored, despite the relevance of black widows to human health and the importance of their venom in studies of vertebrate neurotransmission [33–35]. We identify transcripts with biased expression in the venom gland relative to other tissues, and potential toxin transcripts in the venom gland exome, using bioinformatics-based approaches. We also explore the relative abundance of transcripts specific to the venom gland and quantify the representation of the biological functions and processes in which these transcripts take part. We identify prominent toxin families, and perform phylogenetic analyses to investigate their evolution. Lastly, we explicitly identify the secreted peptide and protein component of the venom using a mass spectrometric based proteomic approach. Our transcriptome and proteome provide complementary data in order to separate the secreted venom components from the cast of molecules that support toxin production within the gland.

## Results

### Bioinformatic functional categorization of the *L. hesperus* venom gland transcriptome

**Figure 1:**
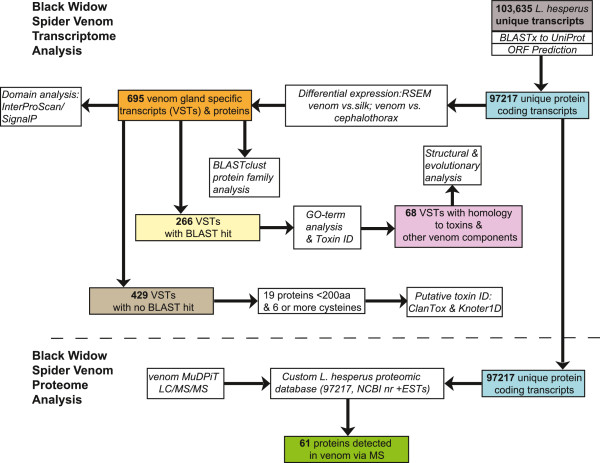
**Flowchart of analyses performed on the set of** ***L. hesperus*** **venom gland specific transcripts (VSTs).** Colored boxes indicate subsets of sequences resulting from specific analyses. Boxes below the dashed line indicate analyses with the combined proteomic and transcriptomic datasets.

RNA-Seq libraries were constructed and sequenced from three *L. hesperus* tissue types: (1) venom glands (52,044,562 reads), (2) silk glands (15,093,424 reads), (3) cephalothorax with venom glands removed (50,969,807 reads). Sequencing reads from each tissue-specific library were separately assembled with Trinity and the three transcript sets were merged with CAP3 [36] to produce a non-redundant set of sequences. This resulted in a transcriptome comprised of 103,635 sequences, 97,217 of which were retained as Unique Assembled Transcripts (UATs) after filtering out sequences encoding identical proteins (see also [37]). Transcripts were submitted to a suite of analyses to investigate their identity, diversity and function in the venom gland, as well as the presence of their products in the venom (Figure 1). Six-hundred ninety-five (0.71%) of the 97,217 transcripts had expected venom gland counts per million (eCPM) greater than one and were either exclusively expressed in the venom gland (386 transcripts), or were among the top 2.5% in the distribution of the ratio of venom gland eCPM values to both silk and cephalothorax. This equates to a level of expression in the venom gland that is at least 306-fold higher than that in silk, and at least 32-fold higher than in cephalothorax. We hereafter refer to this set of 695 sequences as venom gland specific transcripts (VSTs). Of the VSTs*,* 266 (38.3%) had a significant (e-value ≤ 1e-5) BLASTx hit to the UniProt database, while 429 (61.7%) had no significant BLASTx hit to UniProt at this e-value cutoff (Additional file 1). Among VSTs were 45 with significant BLAST similarity to known venom toxins, as well as to 17 enzymes that may act to facilitate toxin action (Table 1, Additional file 1). A total of 1312 GO terms were mapped to 228 sequences in the VST set. GOseq analysis recovered 18 GO terms that were overrepresented in the VSTs compared to all transcripts with an eCPM > 1 at a false discovery rate (FDR) cutoff of 0.05 (Table 2). Overrepresented categories for VSTs in the cellular component ontology included (1) extracellular region, (2) other organism cell membrane and (3) other organism presynaptic membrane. Exocytosis and proteolysis were significantly overrepresented categories in the biological process ontology, while serine endopeptidase and metalloprotease activity were among the overrepresented categories in the molecular function ontology.

**Table 1:** **Summary of groups of toxins and enzymes in** ***L. hesperus*** **venom gland specific transcripts**

Type	Number^a^	% expression	Proteomic evidence	Proposed function
Latrodectins	2	16.1	Yes	Facilitate latrotoxin action
Latrotoxins	39 (20)	15.8	Yes	Form calcium channels, cause neurotransmitter release in target organism
Putative ICK	*7*	14.2	Yes	Potential ion channel action
CRISPs	2/3	8.4	Yes	Calcium channel blocker, smooth muscle paralysis
Metalloproteases	8/11	7.8	Yes	Tissue degradation/spreading factor
ICK toxins	4	5.4	Yes	Alter function of neuronal ion channels
Serine proteases	6/7	2.8	Yes	Tissue degradation/ spreading factor
Chitinases	1	1.6	Yes	Breakdown of arthropod exoskeleton
LRR proteins	8/9	0.82	Yes	Diverse/neural development
Hyaluronidases	2	0.5	Yes	Breakdown of extracellular matrix/spreading factor

^a^The number preceding the slash is the total number of sequences with a BLAST hit to the defined type, while the number after the slash indicates the total number of sequences in this group including additional sequences identified in clustering analysis, but without a top BLAST hit to this sequence type. The number in parentheses indicates the minimum number of putative distinct latrotoxin paralogs. The number of putative ICKs is shown in italic as it is not based on BLAST homology (see text).

For each type, the total number of sequences, % of total expression among all venom gland specific transcripts for all sequences of this type, the proteomic evidence for the presence of the type in venom and the proposed function are shown.

**Table 2:** **Overrepresented GO terms in the** ***L. hesperus*** **venom gland specific transcript set**

GOID	Ontology	Term	p-value
GO:0044218	CC	other organism cell membrane	1.17e-73
GO:0044279	CC	other organism membrane	1.17e-73
GO:0072556	CC	other organism presynaptic membrane	1.17e-73
GO:0005576	CC	extracellular region	7.27e-40
GO:0016021	CC	integral to membrane	4.70e-12
GO:0016020	CC	membrane	2.61e-10
GO:0006887	BP	exocytosis	2.42e-68
GO:0019079	BP	viral genome replication	2.26e-07
GO:0006508	BP	proteolysis	2.96e-07
GO:0080009	BP	mRNA methylation	2.65e-05
GO:0042302	MF	structural constituent of cuticle	3.51e-12
GO:0008237	MF	metallopeptidase activity	9.73e-08
GO:0004222	MF	metalloendopeptidase activity	1.56e-07
GO:0003968	MF	RNA-directed RNA polymerase activity	4.22e-06
GO:0004252	MF	serine-type endopeptidase activity	4.78e-06
GO:0008174	MF	mRNA methyltransferase activity	9.57e-06
GO:0004089	MF	carbonate dehydratase activity	1.62e-05
GO:0004415	MF	hyalurononglucosaminidase activity	1.08e-04

All significant terms at an FDR ≤ 0.05 are shown with brief term descriptions, separated into groups by ontology. CC = cellular component, BP = biological process, MF = molecular function.

Of the 695 VSTs, 414 had at least one protein domain prediction from InterProScan, including 179 sequences with no significant BLAST hit at UniProt. Among all protein domains identified more than five times amongst the VSTs, ankyrin domains were most common, while leucine-rich repeat, low density lipoprotein receptor class A, immunoglobulin, chitin-binding, helix loop helix, latrotoxin C-terminal, venom allergen 5, serine protease and metalloprotease domains also commonly occurred in predicted proteins from the VST set (Additional file 2, Additional file 3).

### *L. hesperus* toxin diversity and evolution

#### Latrotoxins

**Figure 2:**
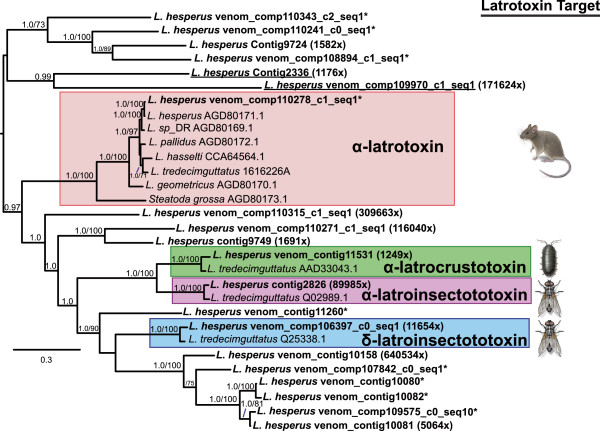
**Phylogenetic tree of latrotoxin protein sequences.** Previously published sequences labeled with NCBI accession numbers and newly assembled transcript sequences from *L. hesperus* with a predicted open reading frame of at least 500 amino acids from this study (in bold). The midpoint-rooted tree is a 50% majority-rule consensus of 3002 trees sampled in Bayesian analysis. Values at nodes show posterior probabilities ≥ 0.95, followed after the slash by ML bootstrap values when >= 70%. Shaded boxes indicate clades of known latrotoxin subtypes associated with specific phyletic targets with representative targets shown to the right; illustrations by Emily Damstra and used here with her permission. An asterisk symbol (*) after the name of the sequence indicates exclusive expression in the venom gland (zero eCPM in other tissues) otherwise the minimum fold difference in expression between the venom gland and the other two tissues is indicated. Underlined sequences vary in placement between the Bayesian and ML trees, as described in the text.

The majority of the diversity among VSTs with BLAST homology to known toxins was contributed by latrotoxins. Strikingly, the number of distinct sequences found suggests a wider range of latrotoxin diversity than previously reported [38, 13]. A total of 39 VST sequences were identified as latrotoxins. Latrotoxins are large proteins, and range from ~1200 to 1400 amino acids in length [19–22], posing a challenge for transcript reconstruction from short-read data. The predicted latrotoxin proteins from the *L. hesperus* transcriptome varied in length, and many appear to be fragments of larger proteins. We aligned the 20 latrotoxin sequences in the set with a predicted protein of at least 500 amino acids with ten additional complete or near full-length latrotoxin sequences from *Latrodectus* and *Steatoda* species from the NCBI *nr* database. A Bayesian phylogenetic tree produced from this alignment included four sub-clades each containing one of the four functionally characterized latrotoxins from *L. tredecimguttatus*, together with conserved orthologs assembled from our *L. hesperus* short-read data (Figure 2). However, the majority of *L. hesperus* sequences we assembled with homology to latrotoxins were not contained within these four clades and were instead dispersed among multiple highly supported clades. The maximum-likelihood topology was identical to the Bayesian tree with the exception of the placement of two sequences (Contig 2336 and venom_comp_1099970_c1_seq1) as unresolved branches at the base of the tree, instead of together in a clade (Figure 2).

**Figure 3:**
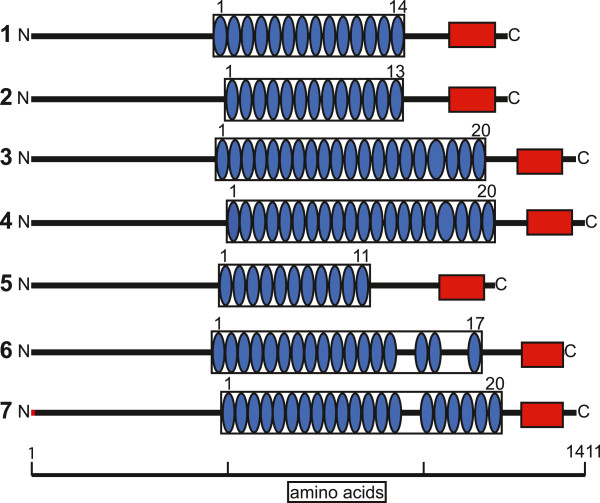
**Representation of domain structure for selected previously published latrotoxins and latrotoxin sequences from this study.** Predictions from InterProScan are shown for ankyrin repeats (blue ovals) and the latrotoxin C-terminal domain (red rectangles). 1 = venom_comp_106397_c0_seq1, 2 = *L. tredecimguttatus* δ-latroinsectotoxin, 3 = Contig2826, 4 = *L. tredecimguttatus* α –latroinsectotoxin, 5 = venom_Contig10081, 6 = venom_comp110241_c0_seq1, 7 = *L. hesperus* α –latrotoxin. The red bar at the N-terminus of sequence 7 indicates 9 amino acids not present in the published sequence that are predicted from the orthologous transcript in this study.

Seven latrotoxin protein sequences inferred from *L. hesperus* VSTs possessed a start codon as well as the distinctive latrotoxin C-terminal domain [38], followed by a 3′ UTR, suggesting that they are full-length or near full-length copies. We examined the domain structure of these putative full-length latrotoxins. Multiple ankyrin repeats (protein-protein interaction motifs with a helix-loop-helix structure [39]) were predicted in each of these sequences, and the number of repeats ranged from 11 to 20 per sequence. Variation was also evident when comparing *L. hesperus* protein sequences with the functionally characterized orthologs from *L. tredecimguttatus* (Figure 3). One sequence, venom_comp106397_c0_seq1 (labeled 1 in Figure 3), groups closely with *L. tredecimguttatus* δ-latroinsectotoxin (sequence 2), but has 14 repeats, as opposed to 13 in the published sequence from *L. tredecimguttatus*[21]. The *L. hesperus* ortholog of α-latroinsectotoxin has 20 ankyrin repeats, as does that of *L. tredecimguttatus*. Other novel *L. hesperus* latrotoxin sequences had either 11 or 17 ankyrin repeats (Figure 3).

We searched the entire translated *L. hesperus* transcriptome to identify other sequences with homology to latrotoxins, but lacking venom gland biased expression. As ankyrin domains are common components of many non-homologous proteins with diverse functions, we limited the BLASTp search to the conserved and distinct N-terminus of the latrotoxin protein, which lacks ankyrin repeats. Two hits were recovered. However, read count data indicate that they lack expression in tissues other than venom gland, and were not included as VSTs because they did not reach the minimum read count threshold for inclusion. These two sequences were not included in phylogenetic analyses, as they did not meet the minimum length requirement.

#### ICK toxins and other small proteins with potential toxicity

**Figure 4:**
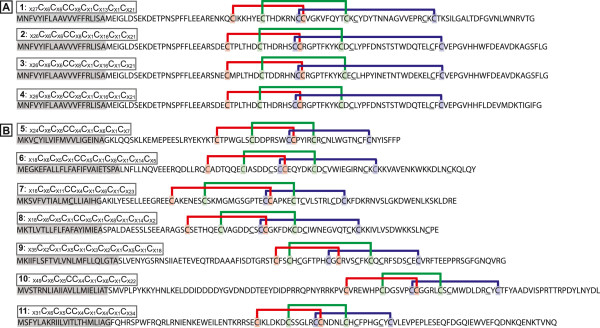
**Amino acid sequences from** ***L. hesperus*** **transcripts containing predicted inhibitory cystine knot (ICK) motifs.** Sequences with BLAST homology to known ICK toxin sequences **(A)** or lacking a BLAST hit but possessing a predicted ICK scaffold **(B)**. The cysteine spacing is numbered by the sequence in the mature toxin. The predicted signal peptide is shaded gray, and the KNOTER1D predicted disulfide connectivity is indicated by colored bars and cysteine residues. Cysteines not predicted to participate in disulfide bonds are underlined. 1=venom_comp104578_c0_seq1, 2=venom_comp104578_c0_seq3, 3=venom_comp104578_c0_seq6, 4=Contig7465, 5=venom_comp72844_c0_seq1, 6=Contig3061, 7=Contig5795, 8=Contig7277, 9=venom_comp98528_c0_seq1, 10=venom_comp75139_c0_seq1, 11= Contig20358.

The most common type of toxin in many spider venoms, as well as in scorpion, cone snail and remipede venoms [40] are small disulfide-bonded neurotoxins with an inhibitor cystine knot (ICK) structural motif. Previously, ICK toxins were not considered to be a part of *Latrodectus* venom, instead potentially being replaced by latrotoxins [14]. However, ICK toxins were recently reported from *L. tredecimguttatus*[13], and our study revealed that sequences encoding these small peptides were also present among the *L. hesperus* VSTs. Specifically, four sequences had BLASTx hits of e-5 or better to members of the spider CSTX toxin superfamily in UniProt. Each of the predicted proteins from these sequences had 8 cysteine residues, and an ICK scaffold predicted by both Knoter1D and InterProScan, containing three inferred disulfide bonds (Figure 4).

**Figure 5:**
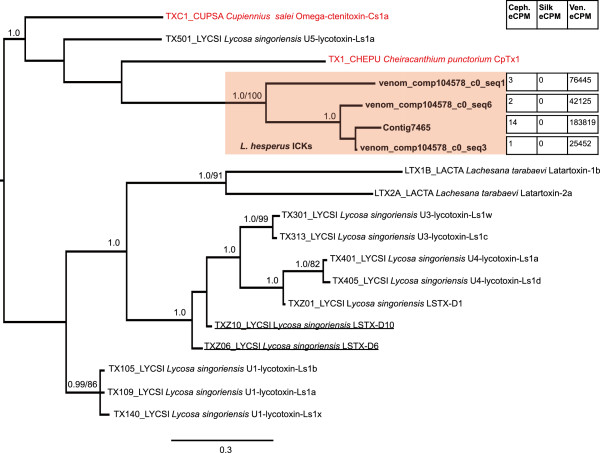
**Bayesian tree of predicted protein sequences from BLAST-identified ICK toxins of** ***L. hesperus*** **and other spiders.** Prefixed identifiers are included for sequences retrieved from the UniProt database. The tree is a midpoint-rooted 50% majority-rule consensus of 3002 trees sampled in Bayesian analysis. Values at nodes are posterior probabilities where they are ≥ 0.95, followed after the slash by ML bootstrap values when >= 70%. Sequences from *L. hesperus* from this study are in bold and the distinct *L. hesperus* clade is shaded in red. Red text delineates sequences for which information is available from prior functional studies (see text for details). Tissue expression levels (eCPM) for sequences derived from this study are shown in chart form (Ceph.=cephalothorax, Ven.=venom gland). The two underlined sequences are flipped in position in the ML tree.

We aligned our four predicted ICK toxin protein sequences (Additional file 4) with 15 sequences retrieved from the ToxProt database [41], representing the range of diversity across the UniProt defined spider CSTX toxin superfamily. The Bayesian and ML trees from this alignment were identical in topology with the exception of two sequences (TXZ10 and TXZ06) that exchanged positions (Figure 5). The *L. hesperus* sequences form their own strongly supported clade (PP = 1.0; 100% bootstrap) within the CSTX superfamily. The most closely related sequences forming a larger clade with the *L. hesperus* ICKs are ICK toxins from three species in the distantly related Superfamily Amaurobioidea [42]. All of these sequences share a conserved 8 cysteine framework [12], but with substantial variation in the number of residues (8–16) between cysteines 6 and 7. Two of the sequences in this clade (Figure 5: omega-ctenitoxin and CpTx1) have demonstrated cytolytic and/or calcium-channel blocking activity [43, 44]. Using the four ICK toxin predicted proteins as queries, a BLASTp search of the full *L. hesperus* transcriptome assembly was also performed to identify related sequences lacking venom gland specificity. This search returned a single hit, which appears to be a fragment of an ICK protein that was expressed exclusively in the venom gland, but did not meet the minimum expression level (>1 eCPM) for inclusion in the venom gland specific set.

Small proteins (<200 amino acids) that are comparatively cysteine-rich (at least 6 cysteines, which are necessary to form the three disulfide bonds that are a defining feature of the ICK fold), are potential candidates for novel ICK toxins. Predicted proteins meeting these criteria are in excess in the VSTs (17.2%) compared to the overall transcriptome (6.6%) considering only proteins with an N-terminal methionine. Of the 32 VST sequences that met this definition, 19 did not have a BLAST hit to UniProt at e-5 or better. Seven of the 19 without a BLAST hit had a predicted ICK scaffold from Knoter1D although none had this scaffold predicted by InterProScan (Table 3, Figure 4). Four of the seven were also strongly predicted as toxins (P2 and P3 categories of ClanTox), while one was categorized as possibly toxin-like (P1). These seven proteins ranged in length from 83 to 124 amino acids, possessed 8–10 cysteines (Table 3), and may represent additional instances of distinct ICK toxins in the *L. hesperus* venom-specific transcriptome, for a total of 11.

**Table 3:** **Summary of putative toxins with no BLAST hit**

ID	Clantox	Knoter1D	Iprscan_domains	#Cys	Length
Venom_comp72844_c0_seq1	P3	Knottin	SP, NC	9	83
Contig3061	P2	Knottin	SP, TM, NC, CC	10	98
Contig5795	P3	Knottin	SP, NC	9	95
Contig7277	P2	Knottin	SP, NC	10	91
Venom_comp98528_c0_seq1	P1	Knottin	SP, TM, NC	10	107
Venom_comp75139_c0_seq1	N	Knottin	TM, NC, CP	8	124
Contig20358	N	Knottin	TM, NC, CP	8	116
Venom_Contig10790	P1	Negative	None	6	65
Contig2158	P1	Negative	None	5	52
Venom_Contig4851	P1	Negative	None	5	64

The ID of each sequence is included, together with ClanTox prediction category, Knoter1D prediction of knottin (ICK) scaffold status, protein domains recognized in InterProScan, the number of cysteine residues and protein length. SP = signal peptide, TM = transmembrane domain, NC = non-cytoplasmic domain, CP = cytoplasmic domain, CC = coiled-coil. P3=toxin-like, P2=probably toxin-like, P1=possibly toxin-like, N=probably not toxin-like.

#### CRISP proteins and enzymes

**Figure 6:**
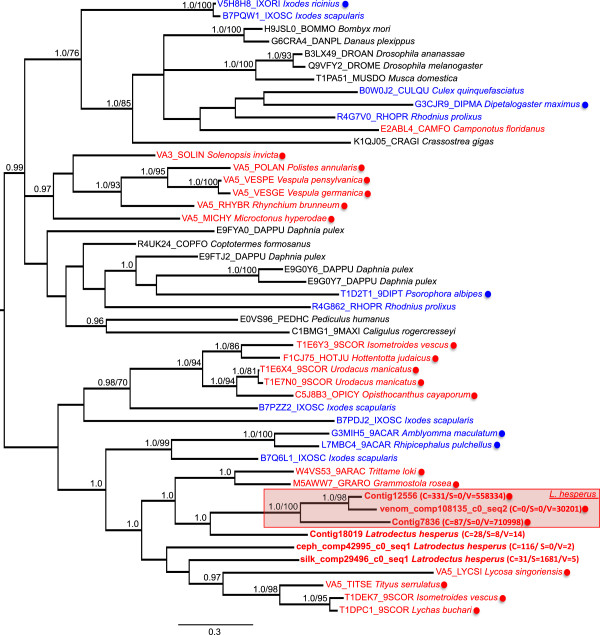
**Bayesian tree of CRISP proteins.** Midpoint rooted 50% majority-rule consensus of 15002 trees. Values at nodes are posterior probabilities where ≥ 0.95, followed by a slash and bootstrap values where ≥ 70% (see also Additional file 5). *L. hesperus* sequences are bold, followed by three tissue expression levels (eCPM) (C = cephalothorax/S = silk gland/V = venom gland). UniProt accession numbers precede species name for other sequences. *L. hesperus* venom gland specific CRISPs are shaded red. Sequences from venomous species in red text, followed by a red dot if venom gland expression is confirmed. Sequences from hematophagous species in blue text, followed by a blue dot if salivary gland expression is confirmed. Sequences from non-venomous/non-hematophagous species in black. *Ixodes ricinius* = castor bean tick, *I. scapularis* = deer tick, *Bombyx mori* = domesticated silkmoth, *Danaus plexippus* = monarch butterfly, *Drosophila* = fruitfly, *Musca domestica* = housefly, *Culex quinquefasciatus* = southern house mosquito, *Dipetalogaster maximus* = kissing bug, *Rhodnius prolixus* = assassin bug, *Camponotus floridanus* = Florida carpenter ant, *Crassostrea gigas* = Pacific oyster, *Solenopsis invicta* = red imported fire ant, *Polistes annularis* = red paper wasp, *Vespula pensylvanica* = western yellow jacket, *Vespula germanica* = European wasp, *Rhynchium brunneum* = potter wasp, *Microctonus hyperodae* = braconid wasp, *Daphnia pulex* = water flea, *Coptotermes formosanus* = Formosan subterranean termite, *Psorophora albipes* = mosquito, *Pediculus humanus* = body louse, *Caligulus rogercresseyi* = sea louse, *Isometroides vescus* = spider hunting scorpion, *Hottentotta judaicus* = scorpion, *Urodacus manicatus* = black rock scorpion, *Opisthocanthus cayaporum* = South American scorpion, *Amblyomma maculatum* = Gulf coast tick, *Rhipicephalus pulchellus* = questing tick, *Trittame loki* = brush foot trapdoor spider, *Grammostola rosea* = Chilean rose tarantula, *Lycosa singoriensis* = spotted wolf spider, *Tityus serrulatus* = Brazilian yellow scorpion, *Lychas buchari* = Buchar’s scorpion.

Two transcripts in the venom gland specific set had BLAST homology to cysteine-rich secretory proteins of the CRISP family, which are common to the venom of numerous species [1], while a third had a top BLAST hit to an uncharacterized protein, but grouped with the CRISP sequences in clustering analysis (see below). We searched the entire *L. hesperus* transcriptome for other closely related sequences with BLASTp (e-value ≤ e-20), but lacking in venom gland biased expression. The *L. hesperus* transcriptome did contain three sequences most highly expressed in the cephalothorax or silk glands with BLAST homology to venom gland specific CRISPs, and with UniProt BLASTx hits to CRISP family proteins. We conducted phylogenetic analysis on the alignment of *L. hesperus* CRISP proteins with sequences from a range of venomous and non-venomous invertebrates that have BLAST homology to the *L. hesperus* CRISPs (Additional file 4). At the largest scale, there were two clades in the Bayesian and ML trees (Figure 6, Additional file 5), which are highly similar with the exception of the placement of a few weakly supported sequences. All sampled spider sequences occur in one clade, together with most other arachnid CRISPs, including those that show evidence of expression in spider or scorpion venom glands. In contrast, some CRISPs with expression in salivary glands of hematophagous ticks are found in the largely arachnid clade, while others appear more closely related to insect CRISPs (Figure 6). The three *L. hesperus* venom gland specific CRISPs form a highly supported clade with a broadly expressed but closely related *L. hesperus* CRISP. Moreover, a number of scorpion venom gland expressed CRISPs group with a copy from the tick *Ixodes scapularis*. Other scorpion CRISPs occur in a different clade with all *L. hesperus* CRISPs, whether venom gland specific or not, along with venom gland expressed CRISPs from other spider species.

Transcripts with homology to several types of enzymes were found in the *L. hesperus* VST set. A total of two hyaluronidases, a single chitinase, and 3 lipases (phospholipase C, AB hydrolase) were identified. A total of 7 distinct serine protease sequences and 8 M13 metalloproteases were found among the 695 in the venom gland specific set. In addition, single sequences with homology to O-sialoglycoprotein endopeptidases and gamma glutamyl transpeptidases were recovered (Additional file 1).

### Clustering analysis of venom-gland specific proteins

**Figure 7:**
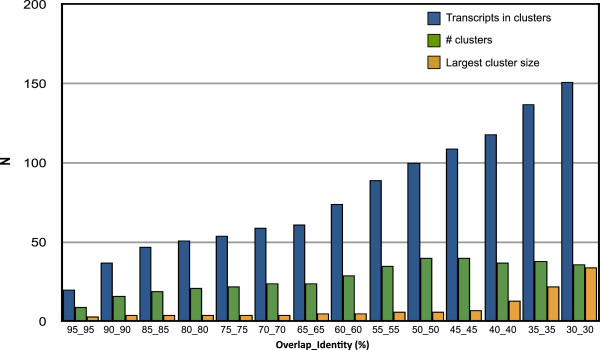
**Results of BLAST-based clustering analysis of** ***L. hesperus*** **predicted proteins from VSTs.** Clustering of sequences was performed across a range of sequence overlap and identity values.

We explored the relationships among VSTs using BLASTclust on predicted proteins to identify putative gene families. Under the most stringent clustering criterion (95% amino acid identity over 95% of the length of both sequences), 675 of 695 protein sequences did not group with any other sequence, and only 9 multiple transcript clusters occurred, with the largest containing 3 members. As the sequence identity was reduced while maintaining stringent (95%) overlap, additional clusters were recovered to a maximum of 20 at ≥ 30% sequence identity. These 20 clusters contained 48 of 695 transcripts, and the largest cluster included only five sequences. Relaxing the percent overlap of sequences while keeping the 95% sequence identity threshold produced a similar result, with 44 transcripts in 19 clusters, and a maximum cluster size of five, at ≥ 30% sequence overlap. When both criteria were relaxed, the number of transcripts in clusters increased rapidly below 65% sequence identity and 65% sequence overlap (Figure 7). The most pronounced increase in cluster size (maximum of 34 members) occurred below 40% overlap and 40% identity, while the number of clusters decreased slightly. At the most permissive threshold for group formation (30% overlap, 30% identity), approximately 22% of transcripts belonged to 36 groups with at least one other member.

There were several clusters with members homologous to known toxins. Under the most permissive clustering criterion, the largest of these groups had 34 members, all but three of which had best BLASTx hits to latrotoxins in the UniProt database, with the other sequences likely clustered due to weak similarity in the ankyrin repeat regions. A second group contained four additional latrotoxin sequences. Membership in the larger group was highly sensitive to the stringency of the clustering parameters, as at 35% overlap and 35% identity, only 22 sequences remained, all with homology to latrotoxins, and at 45% overlap and 45% identity this cluster had fragmented into several smaller clusters, the largest of which contained six members (Additional file 6). The four sequences with homology to ICK toxins also formed a group at the lowest clustering stringency, but this group appeared more coherent: these sequences remained clustered as stringency was increased until 75% overlap at 75% identity was reached.

Other clusters containing more than five members at the most permissive threshold (30% overlap, 30% sequence identity), and representing putative venom gland expressed families, included sequences with homology to cuticular proteins (18 members), M13 metalloproteases (11), leucine-rich repeat (LRR) proteins (7), and serine proteases (6), while the two CRISP proteins identified by BLAST homology clustered with an uncharacterized protein.

### Highly expressed venom gland transcripts

**Figure 8:**
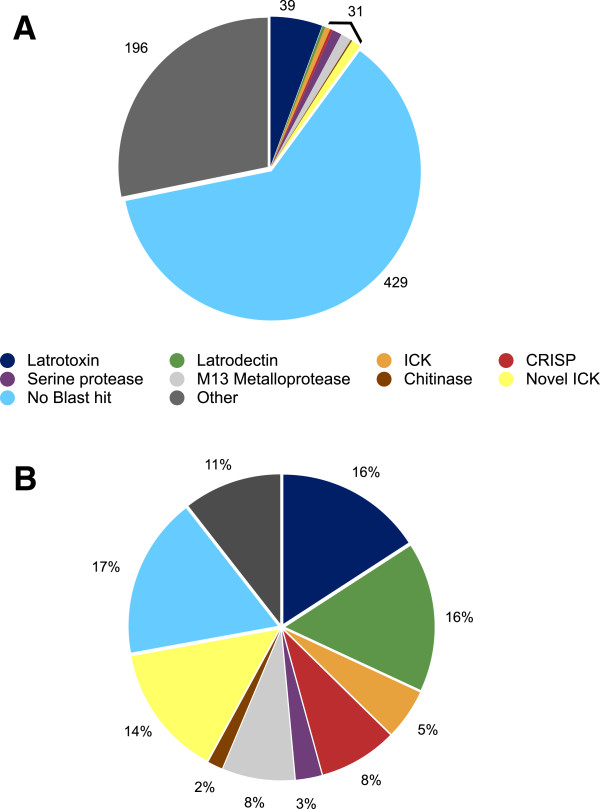
**Summary of diversity and expression of** ***L. hesperus*** **venom gland specific transcripts. (A)** The proportion of total distinct venom gland specific transcripts assigned to several known toxin types or enzymes by BLASTx significant similarity, and assigned to all other categories labeled as “other”, or lacking a significant BLAST hit. The numbers of sequences in the smaller categories were summed for clarity. **(B)** Overall expression as % of total FPKM in the venom gland specific set using these same categories.

Substantial variation in abundance existed among VSTs (Additional file 1). However, a minority of sequences with BLAST homology to known toxins and associated proteins constituted a significant (45.8%) proportion of overall VST expression (Table 1; Figure 8). If the putative ICKs (cysteine-rich small proteins with no BLAST homology but ICK domain predictions) are included this figure rises to 60%. Toxin transcripts were common among the most highly expressed VSTs and included sequences with BLASTx homology to known latrotoxins, ICK toxins, CRISP family toxins, and latrodectins (2 of the 3 most highly abundant VSTs). A number of proteases, as well as a chitinase, were amongst the most highly expressed transcripts, and proteases constitute approximately 11% of the overall expression (Table 1, Additional file 1). In addition, 4 of 7 small, disulfide-rich proteins with no BLAST hit, but with ICK domain predictions, were amongst the most highly expressed VSTs (Table 3, Additional file 1), as were two other small cysteine-rich proteins with no ICK or ClanTox prediction. While only two distinct latrodectin sequences were found, they constituted approximately 16% of the total expression for all venom gland specific transcripts (Figure 8). In contrast, the latrotoxins contribute a similar proportion of transcript abundance (15.8%) but are much richer in sequence diversity, with 39 sequences that likely represent at least 20 paralogs (Figure 8).

### Proteomic and bioinformatic analysis of secreted components

Using Multidimensional Protein Identification Technology (MudPIT) analyses, we identified 61 proteins from an *L. hesperus* protein database that matched peptides collected from *L. hesperus* venom with mass spectrometry techniques (Additional file 7). The analyzed protein database contained 105,520 *L. hesperus* proteins predicted from two transcript sets: (1) 103,635 from the overall *L. hesperus* transcriptome *de novo* assembly, and (2) 483 venom gland ESTs, together with 414 *L. hesperus* proteins from NCBI’s *nr* database. The venom detected proteins included 21 latrotoxins, 1 ICK toxin, and 6 CRISP family toxin proteins (Table 4). Four other sequences from the cysteine-rich set of unknown proteins were also found in the venom, including two that potentially represent additional ICK toxins (Table 3, Additional file 7). Several types of enzymes were identified in *L. hesperus* venom, including hyaluronidases, chitinase, serine proteases and metalloproteases, as were several leucine-rich repeat proteins and three latrodectins (Table 1; Additional file 7). Of the proteins matched, most (49) were predicted from VSTs, while 3 were from transcripts that were venom gland biased, but were not in the upper 2.5% of the distribution of eCPM ratio values. Eight protein sequences predicted from venom gland ESTs, and one from a previous study of latrotoxins [25], were also detected in *L. hesperus* venom.

**Table 4:** **Predicted neurotoxin proteins identified in venom**

ID	Best BLAST hit	# unique peptides	% coverage
Contig14^a^	CRISP	42	51.90%
Contig29^b^	CRISP	5	6.87%
LHV_229_Sp6	CRISP	5	24.30%
LHV_312_Sp6	CRISP	2	3.77%
Contig12556	CRISP	21	38.90%
Contig7836	CRISP	25	44.30%
venom_comp104578_c0_seq1	ICK	2	27.00%
HV209_Sp6_4	α-latrotoxin	2	11.60%
gi|380876959|sp|P0DJE7.1|LITD_LATHE	δ-latroinsectotoxin	3	17.00%
Contig2826	α-latroinsectotoxin	47	35.40%
Contig9724	α-latrotoxin	23	23.50%
Contig9749	α-latrotoxin	43	40.30%
Venom_comp106397_c0_seq1	δ-latroinsectotoxin	32	30.50%
Venom_comp107842_c0_seq1	δ-latroinsectotoxin	15	30.00%
Venom_comp107842_c2_seq1	δ-latroinsectotoxin	3	37.70%
Venom_comp109612_c1_seq1	δ-latroinsectotoxin	4	19.30%
Venom_comp110241_c0_seq1	α-latrotoxin	7	8.98%
Venom_comp110271_c0_seq3	α-latrotoxin	2	6.88%
Venom_comp110271_c1_seq1	α-latrotoxin	28	28.00%
Venom_comp110278_c1_seq1	α-latrotoxin	25	26.60%
Venom_comp110315_c0_seq1	α-latrotoxin	8	25.80%
Venom_comp110315_c1_seq1	α-latrotoxin	14	20.20%
Venom_comp110343_c2_seq1	δ-latroinsectotoxin	3	5.50%
Venom_Contig10080	δ-latroinsectotoxin	8	8.76%
Venom_Contig10081	δ-latroinsectotoxin	4	4.40%
Venom_Contig10082	δ-latroinsectotoxin	4	4.42%
Venom_Contig10158	δ-latroinsectotoxin	6	13.30%
Venom_Contig11531	α-latrocrustotoxin	44	35.90%

^a^Contains EST sequences LHV_167_Sp6, LHV_012_T7, LHV_012_Sp6, LHV_194_Sp6, LHV_167_T7.

^b^Contains EST sequences LHV_250_Sp6, LHV_144_Sp6, LHV_069_Sp6, LHV_069_T7, LHV_511_Sp6, LHV_123_T7, LHV_123_Sp6, LHV_144_T7, LHV_457_Sp6.

The type of toxin identified by the top BLAST hit to each transcript is shown in column 2. Proteomic evidence is displayed in columns 3 (unique peptides mapping to sequence) and 4 (% coverage of predicted protein with mapped peptides).

Approximately 12.5% (87) of protein translations from the *L. hesperus* VSTs possessed a predicted signal sequence. If only the 313 proteins with a putative methionine start codon are considered, this figure rises to 24.9%. Amongst the toxin homologs in this set, none of the predicted latrotoxin proteins contained a typical eukaryotic signal sequence, while four of four ICK toxins, both CRISP toxins, and both latrodectins, contained a signal sequence, as did all seven other potential ICK toxins with no significant BLAST homology. Five proteases (four serine proteases and one metalloprotease) also had a predicted signal sequence. Thirty-six of the 49 predicted proteins from VSTs detected in venom by mass spectrometry contained an M-start, of which 22 (61%) had predicted signal sequences, consistent with their function as a venom component, as opposed to having an intracellular function.

## Discussion

Spiders are the most species-rich clade of venomous metazoans, and it is likely that millions of toxic compounds remain to be identified in their venom [7, 45]. Next generation transcriptomic and proteomic methods, when used in combination, offer a powerful approach to cataloguing and understanding this complexity, as well as its evolution. By applying these methods to *Latrodectus hesperus*, in the context of a multi-tissue expression analysis, we have identified 695 transcript sequences with strongly biased venom gland expression in this species and confirmed the presence of 61 proteins in its venom. The inferred functions of these sequences indicate that the venom of black widow spiders is extremely diverse at the molecular level, and is the product of a complex evolutionary history.

### Molecular diversity in the *L. hesperus* venom gland and functional implications

We found that only 22% of the 695 *L. hesperus* VSTs shared some sequence overlap at the protein level through BLASTclust analyses, implying that a wide diversity of proteins contribute to venom gland function. Nevertheless, we estimated that at least 20 distinct latrotoxin paralogs are expressed in the black widow venom gland, constituting by far the largest gene family in the venom gland specific set of sequences. The latrotoxin proteins predicted from these transcripts were divergent in amino acid sequence and motif organization (Figure 2, Figure 3), and thus it is likely that they represent distinct loci. While seven latrotoxins have been assigned names based on their taxonomic specificity (5 insect-specific, 1 vertebrate-specific, 1 crustacean-specific) in the related species *L. tredecimguttatus*[38], the sequence of only four of these seven functionally characterized latrotoxins are definitively known [19–22]. We identified orthologs of these four functionally characterized latrotoxins in our transcriptome, but have also quintupled the number of sequenced latrotoxin paralogs in *L. hesperus*. While the functionality of these novel latrotoxins is unknown, some of these sequences have best BLASTx hits to the vertebrate-specific α-latrotoxin. Although functional testing is a requirement for confirmation, some of these sequences could represent heretofore unknown vertebrate specific neurotoxins. Such discoveries are significant because vertebrate neurotoxins have important applications in neurophysiological research, considering the fundamental role of α-latrotoxin in deciphering the molecular mechanisms of neurotransmission. The extensive diversity found among the vertebrate receptors of latrotoxins such as neurexins and latrophilins [46–48], suggests that some of these new latrotoxin variants may interact specifically with different receptor isoforms and could play important roles in their characterization. The variable number of ankyrin domains predicted from nearly full-length sequences in this study could contribute to altered functionality, including the ability of latrotoxin monomers to tetramerize, given the role of ankyrin repeats in protein-protein interactions [39].

Small cysteine rich neurotoxic proteins with the inhibitor cystine knot motif dominate the venoms of many spider species [11]. Our BLAST analyses identified four putative ICK toxin sequences amongst the *L. hesperus* VSTs and one was present in the exuded venom. In addition to these ICK toxins, other small cysteine-rich sequences were venom gland specific in expression and some were present in the venom. Some of these toxins may also be ICK toxins as they possess a predicted ICK domain, while others may represent distinctly different molecular scaffolds, although further research is necessary on their structure and function. The presence of both latrotoxins and ICK toxins in *Latrodectus* venom also suggests novel avenues in research as to how small, selective ion-channel toxins may act synergistically with the non-selective cation channels created by latrotoxin pores in the presynaptic membrane [49, 50]. Three additional cysteine-rich proteins with homology to CRISP toxins (or found by clustering analyses) were also strongly biased towards expression in *L. hesperus* venom gland and present in the venom. CRISP family members were also found to be expressed in the venom gland of the related species *L. tredecimguttatus*[13], indicating that this toxin type may be more widespread within the genus.

Among the other venom gland specific transcripts were multiple sequences with homology to proteins with nervous system related functions (Additional file 1). Examples of these included *bruchpilot* from *Drosophila melanogaster*, involved in synaptic plasticity and regulation [51] and neural cell adhesion molecule L1, the *Drosophila* ortholog of which plays a critical role in neural development [52]. L1-type cell adhesion molecules also play a role in presynaptic organization, and often interact with ankyrin repeat containing proteins [53]. Given the importance of the ankyrin repeat-containing latrotoxins in black widow venom, the venom gland biased expression of these transcripts is intriguing, although their links to the action of latrotoxins are speculative at this point. Lastly, eight sequences with homology to leucine-rich repeat (LRR) proteins were also venom gland specific, and a number of these proteins play key roles in neuronal development and maintenance in both invertebrates and vertebrates [54, 55]. These results suggest that homologs of spider proteins involved in neuronal development or function are being co-opted for venom expression, or the potential for molecular mimicry of neuronal proteins by unrelated venom gland expressed sequences.

### Evolutionary diversification of black widow venom toxins

The development of pools of diverse toxin molecules in venom often involves the expansion of gene families [7]. This process can generate large numbers of distinct transcripts and peptides in certain toxin classes. In cone snails, species may produce from 100–300 small ICK peptides known as conotoxins [9]. Conotoxins are notable for their rapid evolution and the extreme divergence among paralogs within a species at the amino acid level [56]. Similarly, sequencing of spider venom gland transcripts has revealed single species ICK toxin libraries containing more than 100 distinct members [12, 57]. While ICK toxin sequences can also differ dramatically among spiders, clades of more closely related sequences also occur in some spider species, and likely represent more recent, species-specific gene family diversification [45]. This may be true in the case of the *L. hesperus* sequences with BLAST homology to known ICKs. Yet, we also found seven additional ICK motif containing sequences, which were more diverse in length, signal sequence and cysteine arrangement, suggesting the recruitment of multiple ICK motif encoding proteins for black widow venom expression.

Latrotoxins, while the most diverse toxin type in this study, as a whole appear to be limited in phylogenetic distribution, and the origins of these toxins are obscure. Only one paralog (α-latrotoxin) has been recognized outside the genus *Latrodectus*, and to date latrotoxins are only known from three genera of Theridiidae [26]. Although repeated ankyrin domains are found in a wide range of unrelated proteins of various functions [58], the latrotoxin N-terminal region appears to be somewhat unique to latrotoxins. A BLASTp search with latrotoxin N-terminal sequences (first 320 amino acids) against the non-venom gland specific *L. hesperus* transcriptome did not find any significant hits. However, we performed a BLASTp search with the *L. hesperus* α-latrotoxin N-terminal region against NCBI’s *nr* database, and found a significant hit to a hypothetical protein from *Diplorickettsia massiliensis* (Accession WP_010598965; e-score 1e-16), an obligate intracellular bacteria isolated from the tick *Ixodes ricinus*, which is a human disease vector. In addition to N-terminal region sequence similarity, the overall length (1286 amino acids) and possession of multiple ankyrin repeats of this bacterial protein are reminiscent of latrotoxins. A recent study by Zhang et al. [59] described similarities between the C-terminal domain of latrotoxins and proteins from arthropod bacterial endosymbionts such as *Wolbachia* and *Rickettsiella*, and suggested that spider latrotoxins were acquired via lateral gene transfer from bacteria. Alternatively, Garb and Hayashi [26] suggested a possible link between latrotoxins and dTRP1a, a *Drosophila* calcium permeable transmembrane channel protein involved in sensitivity to temperature and chemical irritation that contains numerous ankyrin repeats. As genome sequences for *Latrodectus* and related theridiid species become available, these questions regarding the evolutionary origin of latrotoxins may become answerable.

Given the broader phylogenetic distribution of α-latrotoxin outside of *L. hesperus*[26], it will be important to determine if the additional latrotoxins we uncovered have orthologs in closely related species having venom that is less toxic to vertebrates when compared to venom from black widows. Phylogenetic analyses of the latrotoxin family across multiple species may illuminate the ecological adaptations of widow spiders, particularly in terms of understanding the functional utility of latrotoxins for a generalist predator of diverse insects and small vertebrates. Three insect specific latrotoxins previously identified in protein separation studies [38] may be represented in the additional latrotoxins we have recovered, but the functional and taxonomic specificity of the others remains to be determined. Such functional analyses will be necessary to reconstruct whether ancestral latrotoxins have undergone a functional shift from arthropod to vertebrate specificity or vice versa. A comprehensive latrotoxin phylogeny across species could also determine whether gene family expansions are lineage-specific, and correlate with increased venom toxicity and diet breadth.

In contrast to latrotoxins and ICK toxins, the cysteine-rich secretory proteins (CRISPs) are not particularly diverse within the *L. hesperus* VSTs, but we were able to identify three additional transcripts with homology to CRISPs that do not show venom gland specificity. A CRISP phylogeny including diverse venomous, non-venomous and hematophagous arthropods indicates a dynamic evolutionary history for this gene family, with multiple recruitments to function in venom or salivary glands, including a potentially recent CRISP protein recruitment for venom function in *Latrodectus*. A similar conclusion was reached with a less densely sampled, but broader taxonomic selection of CRISPs [1], and more extensive arthropod transcriptomic and genomic resources may identify the gene duplications and changes in tissue-specific expression patterns leading to this pattern.

### Highly expressed transcripts, venom composition and secretory mechanisms

Among the venom gland specific transcript set, overall expression is dominated by putative neurotoxins and their associated molecules, although they make up only a minority of the distinct transcripts. Strikingly, the proportion of transcripts that latrodectins represent is similar to that for all latrotoxin sequences, although latrodectin sequence diversity was at least ten times lower than that of latrotoxins. This suggests that the role of latrodectins in facilitating latrotoxin toxicity may be the same for all latrotoxins, including novel forms identified in this study. Protease expression also accounts for a substantial proportion of VST abundance, and several proteases were amongst the most abundant transcripts in the venom gland specific set.

Proteomic analysis of *L. hesperus* venom also indicates that at least some proteases are secreted, as together with other enzymes (hyaluronidases and chitinase), they were identified in *L. hesperus* venom. Hyaluronidases are found in venom from a range of spider species [14], but whether proteases are an active component of venom in spiders has been a subject of some debate, as some authors argue that protease activity in venom is due to digestive secretion contamination [60]. Our finding of proteases with venom gland specificity, together with the presence of a subset of proteases in the venom, some with predicted secretory signal sequences, may be related to a dual function. Some *L. hesperus* proteases may in fact function in prey immobilization, either acting as toxin spreading factors, or in hemostasis disruption, as is the case in snakes [7, 61], while others may be involved in processing toxin preproproteins into mature toxins [25].

Our mass spectrometry analyses indicated that the majority of the neurotoxin transcripts specific to the venom gland encoded peptides and proteins that were secreted into the venom. Predicted neurotoxins that were not present in collected venom may reflect the variability inherent in venom-related gene expression, as data acquisition for the transcriptome and proteome was performed on different individuals. It may also reflect variation in the processes of translation or secretion among individual spiders. Overall, the limited number of venom gland specific genes whose products are found in the venom itself is rather unexpected, given the purported mechanism of *L. hesperus* secretion into the venom gland lumen, in which the secretory cells disintegrate and expel the entirety of their contents [23, 62]. Yet there would appear to be some filtering mechanism that is selective against most proteins from VSTs, as few appear in the venom itself. The possession of a signal sequence may constitute such a filter. While only a minority (25%) of complete predicted proteins from VSTs have a predicted signal sequence, the majority of proteins (67%) identified in the venom by mass spectrometry have predicted signals. Latrotoxins seem to be an exception, lacking a typical eukaryotic secretion signal, yet being common in the venom itself. However, previous work has indicated the presence of a cleaved sequence on the N-terminus that could potentially function as a non-canonical secretory signal [21].

## Conclusions

In this study, next-generation RNA sequencing of multiple tissues coupled to proteomics has provided a wealth of insight into venom gland expression and the molecular complexity of *Latrodectus* venom. Numerous new variants of known toxins were identified, and potentially novel toxins of unknown function recovered, suggesting the need for a fundamental reconsideration of the functional activities of black widow spider venom in natural prey and in human envenomation. The extreme pain associated with black widow spider bites is typically accompanied by additional symptoms (e.g., diaphoresis, hypertension, paresthesia, fasiculations [63]), which in addition to α-latrotoxin, may be caused by other toxins uncovered in this study. This expanded toxin library can also be mined for novel molecular probes or drug leads. Of particular interest for neurophysiology is the large number (≥20) of previously unknown latrotoxin variants and 11 ICK motif containing proteins discovered in this study, which may offer new avenues for dissecting the molecular mechanism of neurotransmitter release and for characterizing neuronal ion channels. These functionally diverse latrotoxins comprise a large venom gland expressed gene family with a highly restricted phylogenetic distribution, suggesting they have undergone a rapid evolutionary expansion in black widow spiders.

## Methods

### *L. hesperus* transcriptome sequencing and assembly

Paired-end Illumina sequencing was performed by the Genomics Core at the University of California, Riverside, on cDNA libraries generated using the Illumina mRNA sequencing sample preparation kit with mRNA from three tissue types: (1) venom gland, (2) silk glands and (3) cephalothorax minus venom glands, each in a single lane [37]. After trimming of adapters and low quality sequence, reads from each individual library were separately assembled using Trinity [64], and subjected to CAP3 [36] to merge transcripts under default parameters and reduce redundancy in the transcript set, producing contigs with the tissue type as a prefix (i.e. venom_Contig0000). CAP3 was then applied a second time to merge transcripts across tissue-specific assemblies and produce a set of contigs with no prefix (i.e. Contig0000) as well as retaining contigs from the tissue specific CAP3 assemblies with a tissue-specific prefix, together with non-merged transcripts that retain the original Trinity nomenclature (i.e. venom_comp00000_c0_seq0) with a prefix indicating their tissue origin [65]. All sequences were screened for homology to the UniProt database using BLASTx with an e-value cutoff of 1e-5. Open reading frames (ORFs) for all transcripts were predicted in all six frames using GetORF, filtering out ORFs less than 90 bp in length. A best protein prediction for each contig was generated with a custom Perl script by (1) extracting the longest reading frame in the same frame as the best BLASTx hit, or (2) by extracting the longest reading frame for contigs lacking a BLASTx hit. However, proteins with a methionine start codon were selected if bounded by stop codons on the 5′ and 3′ ends, indicating the potential for a full-length ORF, and if the M-start ORF was at least 75% of the longest predicted ORF.

After CAP3 assembly at the nucleotide level some transcripts that produced identical amino acid sequences persisted in the data set. Hence we further filtered the transcript set to produce a non-redundant set of proteins and their associated nucleotide sequences. BLASTclust [66] was employed to identify sets of protein sequences in which members were identical over their entire region of overlap. In cases in which proteins varied in length within a cluster, all but the longest member of the cluster was removed from both the protein and nucleotide sequence libraries using a custom Perl script. Otherwise, the first member was arbitrarily chosen to represent that cluster.

### Identification of venom gland specific transcripts

To identify venom gland specific transcripts (VSTs), RSEM [67] was used to estimate transcript abundances by mapping reads from the venom, cephalothorax and silk libraries against the assembled and filtered non-redundant transcriptome using Bowtie with default parameters [68]. Expected read counts per million (eCPM) in each tissue for each transcript were calculated and the distribution of the log of the ratio of eCPM of venom gland to silk and venom gland to cephalothorax for each transcript was plotted. Transcripts for which venom gland expression of greater than one eCPM was observed, with zero eCPM in the other two tissues, were identified. Further VSTs were identified as those with a ratio of venom eCPM/silk eCPM and venom eCPM/cephalothorax eCPM in the upper 2.5% of the distribution of the remaining transcripts, and at least one eCPM in venom. Together, transcripts from these two categories constitute the venom gland specific set. Fragments per kilobase per million reads (FPKM) values were also calculated in RSEM for comparing abundances amongst VSTs.

### Functional analysis of venom gland specific transcripts

GO terms were retrieved from UniProt-GOA for the best BLASTx hit to each sequence and used to annotate the *L. hesperus* sequence set. Additional GO terms were mapped by searching the Pfam-A database for sequence homology to predicted protein sequences using the probabilistic Hidden Markov models implemented in HMMER 3.0 [69].

To correct for potential transcript length bias in differential expression in RNA-Seq experiments, GOseq [70] was performed to find overrepresented gene ontology categories in the set of venom gland specific transcripts to identify biological processes and functions important in the venom gland. This method corrects for the violation of the assumption that all genes are equally likely to be identified as differentially expressed, an assumption that does not hold for read count based methods such as RNA-Seq, and the violation of which causes false positives for categories with an excess of long genes in GO overrepresentation analysis.

### Identification of toxins in the venom gland specific set

Sequences with homology to known toxins were identified in the UniProt BLASTx results using text searches. We identified the potential presence of families of toxin and other transcripts specifically expressed in the venom gland of *L. hesperus* by clustering predicted protein sequences using the BLASTclust algorithm under both permissive and stringent criteria. The BLASTclust output was parsed with a custom Perl script to calculate group sizes, group numbers and group composition by appending BLASTx results.

InterProScan [71] was used on predicted proteins to identify the domain architecture of gene products. ClanTox [72] was used to predict the potential toxicity of translated proteins. The algorithm used takes into account features of the frequency and distribution of cysteine residues in the primary sequence from known peptide toxins [73]. ClanTox produces four categories of toxin predictions based on statistical confidence ranging from N = probably not toxin-like to P3 = toxin-like. Knoter1D was used to predict the connectivity of inhibitor cystine knot structures (also referred to as knottins) from the primary sequence of peptides and proteins [74]. Given that toxins function within an extracellular secretion, predicted proteins were scanned for the presence of a signal sequence indicating targeting to the secretory pathway using SignalP 4.1 [75].

### Venom collection and mass spectrometry

We determined the proteins present in the venom of *L. hesperus* by collecting venom exuded by anesthetized adult females subject to electrostimulation with a 10 V current via a capillary tube, and subsequently diluting the venom in 5 μL of distilled water. The trypsin-digested diluted venom was analyzed by MudPIT analysis [76], performed by the Arizona Proteomics Consortium at the University of Arizona. This method uses a multidimensional liquid chromatography separation followed by tandem mass spectrometry (LC-MS/MS) and the Sequest algorithm [77] to identify digested peptides in *L. hesperus* venom secretions. Scaffold software (Proteome Software, Portland, Oregon) was then used to map peptides found in venom to the predicted protein sequences from the *L. hesperus* assembled transcriptome, together with *L. hesperus* venom gland ESTs, and all *L. hesperus* protein sequences available at NCBI, to identify secreted products. Only sequences with protein and peptide probabilities in excess of 95%, and with at least two mapped unique peptides were considered as present in venom.

### Phylogenetic analysis

Alignments of amino acid sequences were constructed with the COBALT [78] web server at NCBI using default settings for gap penalties and query clustering, and with RPS BLAST enabled. Alignments were trimmed manually or with trimAl 1.2 [79] using the automated1 setting to remove regions with an excessive amount of missing data or poorly aligned regions. Phylogenetic trees were constructed for members of specific gene families using Bayesian analysis of amino acid sequences in Mr. Bayes 3.2.2 [80] sampling across fixed amino acid rate matrices. Two simultaneous runs of 1,000,000-5,000,000 generations using a single Markov chain were performed. Convergence was achieved in all analyses as determined by an average standard deviation of split frequencies < 0.01, effective sample sizes for all parameters > 100, and potential scale reduction factors for all parameters of approximately 1. The first 25% of trees sampled were discarded as burn-in and a 50% majority rule consensus was constructed for each analysis using posterior probability (PP) as a measure of clade support. Maximum-likelihood trees for the same set of gene families were found using RAxML [81] using the BLOSUM62 substitution rate matrix with gamma distributed rate variation among sites. 1000 bootstrap pseudoreplicates were performed to assess support for clades.

### Availability of supporting data

All reads and the final transcriptome described in the manuscript are available under BioProject accession PRJNA242358. Illumina sequence reads have been deposited at NCBI’s SRA archive under the following numbers (Venom: Sample: SAMN2720862, Experiment: SRX512000, Reads: SRR1219652; Cephalothorax: Sample: SAMN2708870, Experiment: SRX511999, Reads: SRR1219650; Silk: Sample: SAMN2720861, Experiment: SRX512001, Reads: SRR1219665). Venom gland ESTs are available under NCBI accession numbers JZ577614-JZ578096 [82].

## Electronic supplementary material

Additional file 1: **A supplemental table that contains expression as FPKM for all transcripts in the venom gland specific set together with top BLAST hit for identification, and expected counts per million (eCPM) for each transcript across the three tissues.** (XLSX 60 KB)

Additional file 2: **A supplemental table showing a summary of the representation of protein domains identified by InterProScan from four major databases (SMART, Pfam, ProSite, PRINTS).** (XLSX 36 KB)

Additional file 3: **A supplemental table that contains all InterProScan predictions on the set of 695 venom gland specific proteins.** (XLSX 319 KB)

Additional file 4: **COBALT alignments of ICK and CRISP proteins.** (PDF 442 KB)

Additional file 5: **Maximum-likelihood tree of CRISP proteins.** (PDF 144 KB)

Additional file 6: **A supplemental table that contains detailed results of cluster membership at each level of stringency indicated in Figure** 7 . (XLSX 50 KB)

Additional file 7: **A supplemental table that shows the results of proteomic analysis, including all predicted proteins from transcriptomic and previously published data that were identified in venom by MudPIT analysis, and salient information about the protein, including whether it had a methionine start and predicted signal peptide.** (XLSX 41 KB)

## Abbreviations

RNA-seq: RNA sequencing
VST: Venom gland specific transcript
kDa: Kilodaltons
ICK: Inhibitor cystine knot
CRISP: Cysteine-rich secretory protein
eCPM: Expected counts per million
MudPIT: Multidimensional protein identification technology
EST: Expressed sequence tag
cDNA: Complementary DNA
ORF: Open reading frame
FPKM: Fragments per kilobase per million reads
GO: Gene ontology.
