# Supplementary material for: Dramatic expansion of the black widow toxin arsenal uncovered by multi-tissue transcriptomics and venom proteomics
Source: BMC Genomics. 2014 Jun 11;15(1):366. doi: 10.1186/1471-2164-15-366 (PMC4058007; doi:10.1186/1471-2164-15-366)
Supplement: Supplementary file 4 — Additional file 4: COBALT alignments of ICK and CRISP proteins. (PDF 442 KB) [file 12864_2013_6108_MOESM4_ESM.pdf]

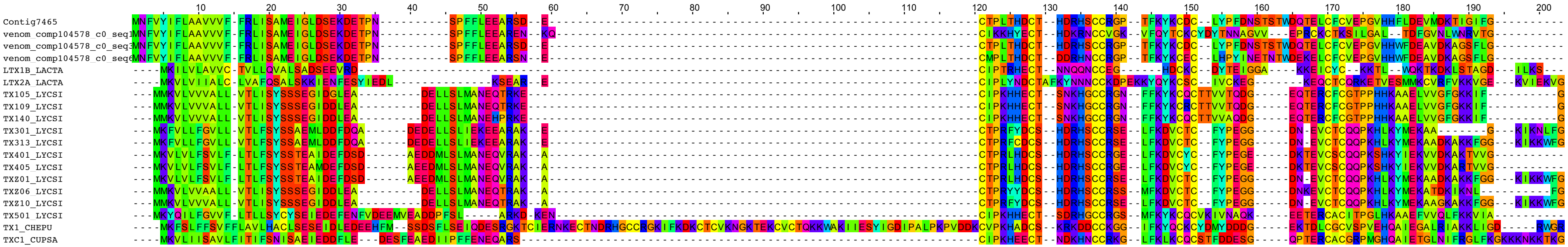

Alignment of ICK toxin proteins used in phylogenetic analysis with Taylor color scheme for amino acids (Taylor W., 1997, Protein Engineering 10: 743-746.)

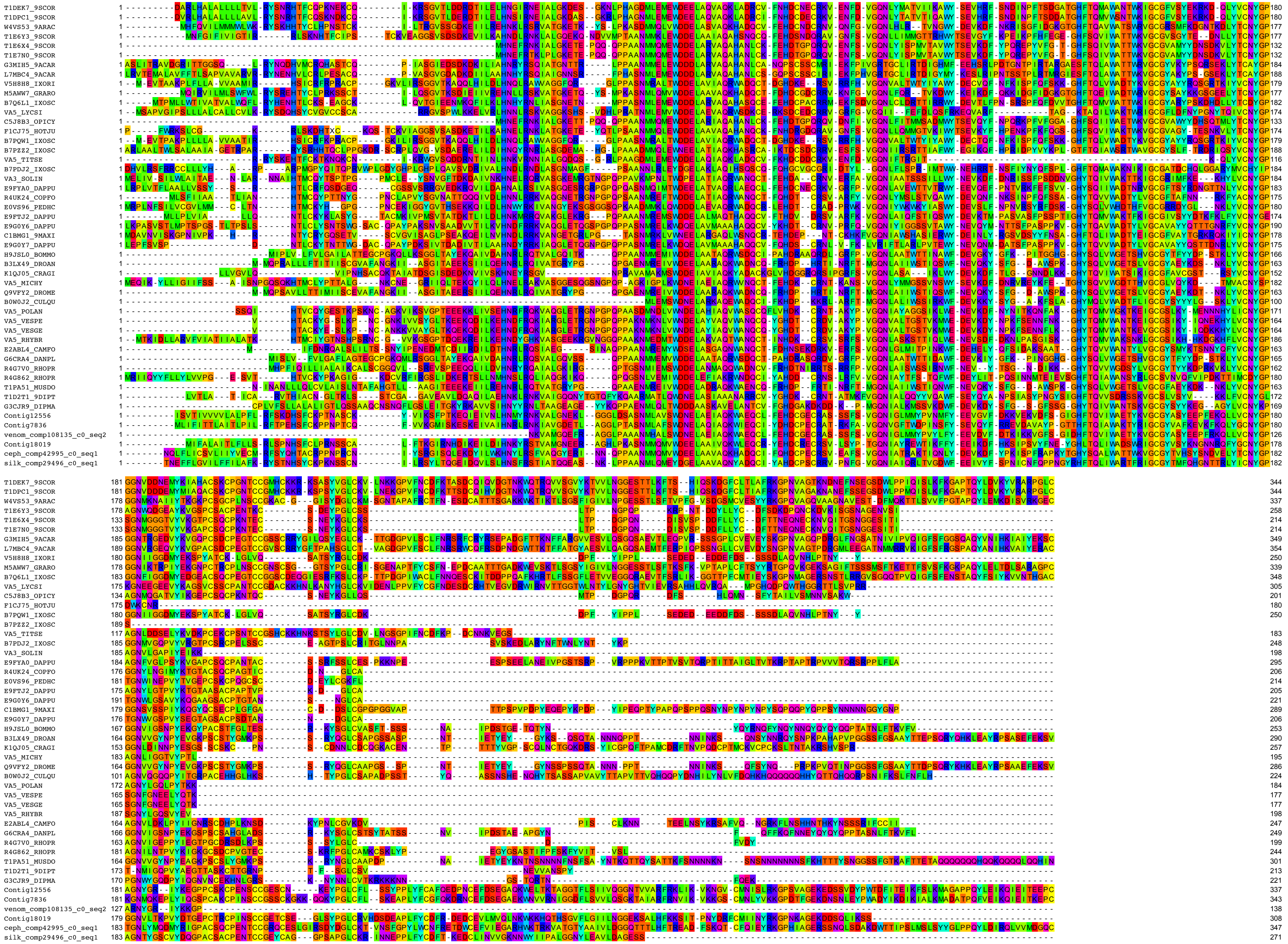

Alignment of CRISP proteins used in phylogenetic analysis with Taylor color scheme for amino acids (Taylor W., 1997, Protein Engineering 10: 743-746.)
