# Supplementary material for: Dramatic expansion of the black widow toxin arsenal uncovered by multi-tissue transcriptomics and venom proteomics
Source: BMC Genomics. 2014 Jun 11;15(1):366. doi: 10.1186/1471-2164-15-366 (PMC4058007; doi:10.1186/1471-2164-15-366)
Supplement: Supplementary file 5 — Additional file 5: Maximum-likelihood tree of CRISP proteins. (PDF 144 KB) [file 12864_2013_6108_MOESM5_ESM.pdf]

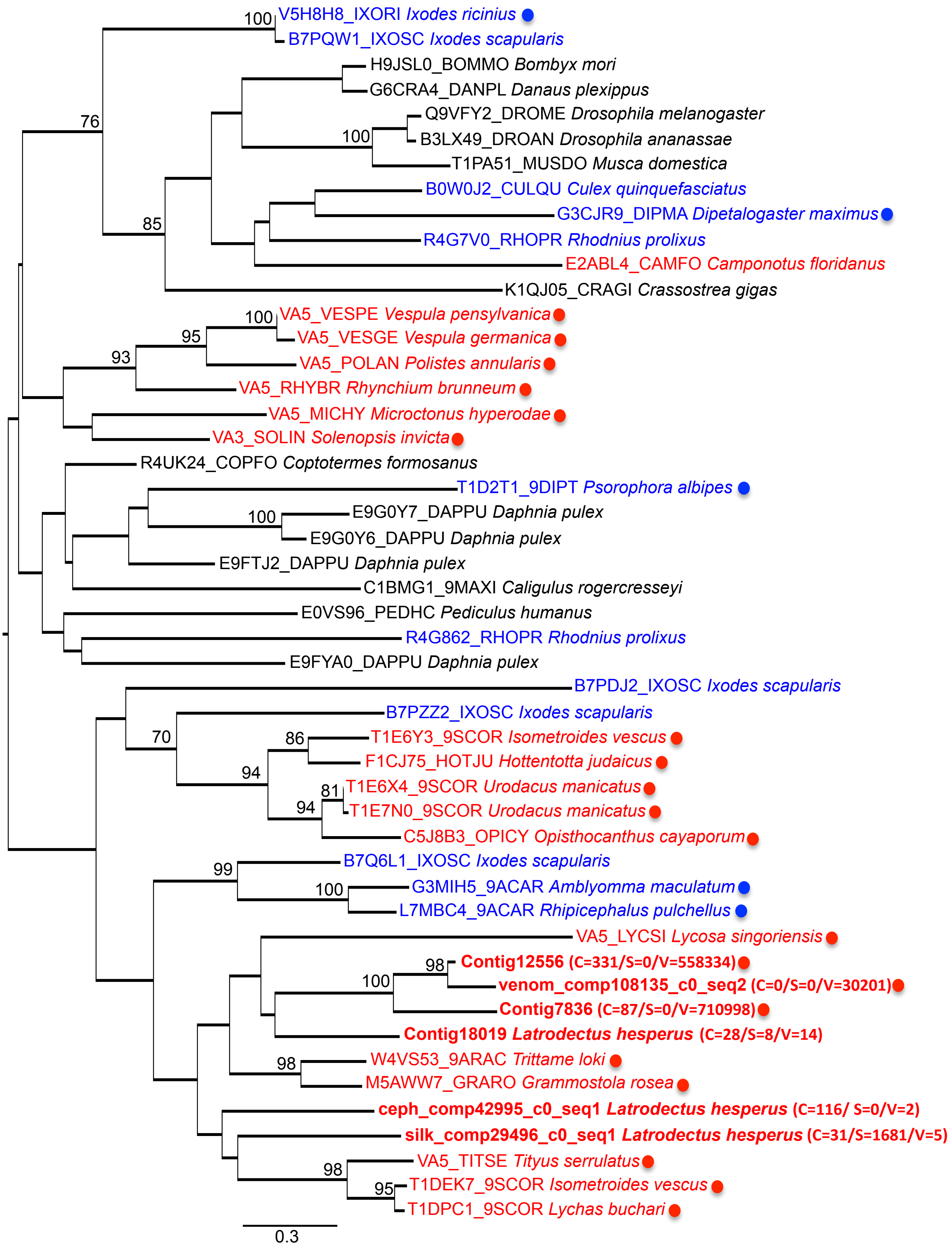

Midpoint rooted maximum-likelihood tree of CRISP proteins. Numbers at nodes are bootstrap values if greater than 70%. Sequences from *L. hesperus* from this study are in bold, with expression levels (eCPM) in the three tissues (C=cephalothorax/S=silk gland/V=venom gland) following the identifier. Other sequences have UniProt accession numbers preceding the species name. Sequences from venomous species are in red text, followed by a red dot if expression of that sequence in the venom gland is confirmed. Sequences from hematophagous species are in blue text, followed by a blue dot if expression of that sequence in the salivary gland is confirmed. Sequences from non-venomous/non-hematophagous species are in black.
